# Vitamin D and epilepsy: are anti-epileptic drugs a double-edged sword? Perspective from low- and middle-income countries

**DOI:** 10.1186/s42506-024-00152-0

**Published:** 2024-03-05

**Authors:** Zain Ali Nadeem, Aimen Nadeem

**Affiliations:** 1 Department of Medicine, Allama Iqbal Medical College, Lahore, 54000 Pakistan; 2 Department of Medicine, King Edward Medical University, Lahore, Pakistan

To the Editor

We know that epilepsy is a widespread neurological disorder. The Global Burden of Epilepsy Report estimated that around 50 million people suffer from epilepsy globally; more than 80% of epilepsy-related deaths are recorded from middle- and low-income countries [1]. An intricate correlation exists between epilepsy and vitamin D. Hypovitaminosis D, via multiple mechanisms, leads to uninhibited electrical discharges in the brain. Furthermore, the commonly used anti-epileptic drugs — particularly enzyme-inducing anti-epileptic drugs (EIAEDs) — may increase the risk of vitamin D deficiency, exacerbating epilepsy instead [2].

Vitamin D deficiency is particularly high in middle- and low-income countries [3]. Over half of the Pakistani population is deficient in vitamin D [4]. In countries where vitamin D deficiency is endemic, imprudent use of EIAEDs further worsens the quality of life of patients with epilepsy. A recent study reported significantly lower vitamin D levels in patients taking valproic acid compared to the healthy control group [5]. The biochemical mechanism aside, various sociocultural factors also precipitate the crisis. Epilepsy may impair social cognition [6], forcing patients to spend more time indoors and limiting their exposure to the sun. The cultural obsession with lighter and untanned skin in some of these countries [7] has created an inclination to avoid the sun, predisposing the population to vitamin D deficiency; this effect is further compounded by EIAEDs. Bone pain and muscle weakness, depression and fatigue, and stunted growth and development in children are common clinical features of vitamin D deficiency [8]. These comorbidities could lead to a sense of hopelessness in patients and thus deteriorates their mental health and their motivation to recover.

Physicians should screen patients with epilepsy for vitamin D deficiency. Admittedly, regular testing might not be feasible on a large scale because of financial constraints; however, high-risk patients should be regularly monitored. A vitamin D-rich diet and supplementation will benefit severely ill patients with limited access to sunlight [9].

Various higher-income countries have devised recommendations regarding vitamin D deficiency, but only a few among the middle- and low-income countries have reached that milestone [10]. Realizing the gravity of the situation, the governments and healthcare community should collaborate to develop an appropriate protocol tailored to their particular requirements. In the meantime, we should ensure that the greater number of healthcare professionals in vulnerable regions are up to date with the currently accepted guidelines. It is imperative that physicians are able to recognize high-risk groups — for instance, patients taking EIAEDs — and judiciously administer suitable therapy, with special focus on prevention. Lastly, physicians and other influential figures should promote confidence and self-esteem in patients with epilepsy and educate them regarding the importance of adequate sun exposure for a healthy life.

## Data Availability

Not applicable.

## Abbreviation

EIAED: Enzyme-inducing anti-epileptic drugs
